# Combined Real-Time Three-Dimensional Hysterosalpingo-Contrast Sonography with B Mode Hysterosalpingo-Contrast Sonography in the Evaluation of Fallopian Tube Patency in Patients Undergoing Infertility Investigations

**DOI:** 10.1155/2019/9408141

**Published:** 2019-06-03

**Authors:** Sumin Chen, Xiya Du, Qingzi Chen, Shaoqi Chen

**Affiliations:** ^1^Department of Ultrasonography, First Affiliated Hospital of Shantou Medical College, 57 Changping Road Shantou, 515000 Guangdong, China; ^2^Department of Ultrasonography, Longhu People's Hospital, 18 Rongjiang Road Shantou, 515000 Guangdong, China

## Abstract

**Objective:**

This prospective study aimed to investigate the use of real-time three-dimensional hysterosalpingo-contrast sonography (4D-HyCoSy), using contrast agent SonoVue, with B mode hysterosalpingo-contrast sonography (B mode-HyCoSy), to evaluate tubal patency and the wall of the Fallopian tubes in infertility patients.

**Method:**

In total, we recruited 739 women with fertility requirements from the First Affiliated Hospital of Shantou Medical College between January 2017 and July 2018. All cases received 4D-HyCoSy using contrast agent SonoVue, immediately followed by the B mode-HyCoSy. Of these patients, 145 showed pathological findings in the Fallopian tubes during HyCoSy; 34 of these (62 Fallopian tubes) were verified by laparoscopy and the dye test against routine reference standards. Sonographic findings, along with laparoscopic findings and dye test results, were used to compare the two techniques using the Cohen kappa coefficient. We also investigated the duration of examination and pain score.

**Results:**

Compared with laparoscopy and the dye test, the tubal occlusion diagnostic accordance rates for 4D-HyCoSy were 88.7% (32+23)/62, with a kappa coefficient of 0.769 and a 76.9% agreement rate. Distal occlusion diagnostic accordance rates for 4D-HyCoSy were 100% (8/8) with a k coefficient of 1.000 and a 100% agreement rate.

**Conclusions:**

The use of 4D-HyCoSy, with B mode-HyCoSy, for the diagnosis of tubal patency is safe, feasible, noninvasive, and highly accurate. B mode-HyCoSy allowed us to observe tubal walls in an intuitive manner.

## 1. Introduction

While there are many reasons underlying infertility, tubal factors remaining are known as a significant cause of female infertility. Obstruction of the Fallopian tubes is responsible for infertility in at least 30% of female cases [1]. Therefore, evaluating tubal patency plays a major role in diagnosing this condition. Traditionally, the techniques used to evaluate tubal patency include hydrotubation, X-ray hysterosalpingography (HSG), laparoscopy, and the dye test. However, these techniques have their disadvantages. Hydrotubation is rarely used now because it is used blindly and has poor accuracy. On the other hand, HSG shows high accuracy (83%) in the diagnosis of tubal patency. However, on the other hand, HSG produces radiation exposure and is associated with potentially allergenic agents [2, 3]. Laparoscopy and the dye test are widely regarded as the current gold standard because of its intuitive approach and high accuracy. However, this technique is expensive, invasive, and associated with anesthetic and surgical risks [4].

Ultrasound techniques and the use of contrast media are the two essential aspects of evaluating tubal status by sonohysterography. Several contrast agents have been used previously, including Echovist, hydrogen peroxide, and saline/gas mixture. Again, these all have their own limitations. Echovist may cause galactose allergies due to its galactose preparation; furthermore, it only has a short effect; within 5 minutes, its dissolution may result in poor visualization [5]. Because of its strong oxidizing ability, hydrogen peroxide may cause irritation and impair the mucosa. Finally, saline/gas mixture is notoriously difficult to distinguish from the surrounding bowels, is easily dispersed, and readily adheres to the pelvic organs.

In the present study, we used the second-generation ultrasound contrast agent, SonoVue. This is a suspension of stabilized sulfur hexafluoride (SF6) microbubbles which provide high resistance. These media are able to respond to ultrasound insonation, at low acoustic pressure, with higher frequency of the ultrasound beam. Research has shown that, as a contrast agent, SonoVue was tested on over 100 000 patients and was regarded as being safe [6]. In 1981, Nannini et al. [7] were the first to introduce HyCoSy to assess tubal patency. HyCoSy has been reported to be as reliable as the laparoscopy dye test or hysterosalpingography in the assessment of tubal patency and uterine abnormity [8–10]. Moreover, some studies have shown that HyCoSy is better than HSG in diagnosing tubal occlusion [11]. Recent years have seen the development and application of three-dimensional hysterosalpingo-contrast sonography (3D-HyCoSy), along with appropriate new forms of contrast media [9, 12, 13]. This is a single volume data imaging modality, which is capable of visualizing the morphology of the uterine cavity and the shapes of the entire Fallopian tube, thus providing an abundance of information to evaluate. The new generation of this technique, 4D-HyCoSy, uses multiple volumes under low mechanical indices and harmonic imaging, to reconstruct the dynamic observation of two-dimensional (2D) ultrasound and present this in a volume of three-dimensional (3D) ultrasound data. Using this technique, it is possible to show the uterine cavity morphology and entire Fallopian tube, even if the tube is tortuous or angled; it is also possible to observe the movement of contrast media flowing through the Fallopian tubes and the pelvis [14, 15].

In this study, we used 4D-HyCoSy, with B mode-HyCoSy, to explore the clinical value of this technique for the evaluation of tubal patency. B mode-HyCoSy is a non-Doppler method which is capable of displaying fallopian tube lumen. The key aspect of our study is that 4D-HyCoSy may readily visualize the entire Fallopian tube, even it is tortuous or angled, while B mode-HyCoSy can observe tubal walls in a highly effective manner.

## 2. Materials and Methods

### 2.1. Research Subjects

This prospective study was undertaken in the First Affiliated Hospital of Shantou Medical College between January 2017 and July 2018 as part of our female infertility program. In total, 739 female patients were recruited; these were 21–44 years of age with a mean infertility duration of 3.2 ± 2.1 years. The study protocol was approved by the Institutional Review Board of the First Affiliated Hospital of Shantou Medical College. The purpose of the study was explained to all patients in detail and they signed consent prior to recruitment. The examination was conducted during days 5–12 of the menstrual cycle [16]. The patients included in this study had no history of serious diseases and contraindications. Each patient underwent bacteriological screening of the cervix before the procedure. Patents with irregular vaginal bleeding were tested to eliminate an early pregnancy and were given progesterone treatment prior to examination.

### 2.2. Instruments

All examinations were performed with a Samsung WS80A with an Elite, Samsung color Doppler Ultrasound, and a transvaginal 5-9 MHz transducer with 2D, 3D, and 4D capabilities. SonoVue was used as the ultrasonic contrast agent (Bracco, Italy); the dry powder contrast agent was diluted to 5.0ml with 0.9% chlorine sodium solution. After sufficient shaking, 2.5 mL of the microbubble suspension was extracted and dissolved in 17.5 mL of 0.9% sodium chloride solution.

### 2.3. Methods

Patients were positioned in the lithotomy position, a speculum was inserted, and the vagina and cervix were disinfected with a 10% povidone-iodine solution. Sterile draping was applied and a Foley catheter no. 12 (Jiangyang, Ltd., Yangzhou, Jiangsu, China) was inserted into the cervical canal. Then, 1.5mL of 0.9% sodium chloride solution was inserted into the balloon to ensure that the cervical canal was closed and the catheter located appropriately. Then, the speculum was removed and a transvaginal probe was inserted into the posterior vaginal fornix. Sterile saline was initially injected into the uterine cavity using 2D and 3D HyCoSy to evaluate any abnormal findings of the uterus and ovaries. We also attempted to recognize the uterine horn on the coronal plane using 3D HyCoSy. Then, the two diagnostic procedures in our study were performed sequentially. We switched to four-dimensional mode and prescan by using a 2D fan angle of 180° and a 4D sweep angle of 90°. We used this system to investigate the uterus, ovaries, and pelvic cavity; image quality was set to maximum. If the bilateral ovaries were too far from each other to be included in the max sweep angle, we would rescan the tubes separately to ensure that the entire Fallopian tubes were included in our imaging. Then, we injected the SonoVue slowly and evenly until the uterine cavity was fully expanded. Then, we observed the uterine cavity and the beginning of the bilateral tubes, which were filled with contrast agent and used to track the flow to the fimbria and pelvic cavity. The acquired data were stored by pressing P2 for offline analysis and reconstruction. When 4D-HyCoSy had been completed, we immediately converted to the B mode-HyCoSy procedure. Two skilled sonographers independently analyzed the data; if a consensus was not reached, a third sonographer was consulted. The duration of the examination and pain score were evaluated during and after the examination. All patients were given antibiotic treatment for 2 days to prevent infection.

### 2.4. Diagnostic Criteria for 4D-HyCoSy [14, 17]

1. Tube patent: 4D reconstruction revealed no resistance and reflux. The contrast agent flowed from the uterine cavity into the uterine cornu and through the Fallopian tube and finally arrived at the fimbriae end of the tube. The passage of the tube was natural and smooth. An annular high echoic area was evident around the ovaries, and microbubbles were dispersed evenly within the pelvic cavity.
2. Tube patent but not smooth: 4D reconstruction revealed mild resistance and reflux. Pressurized infusion was needed. Contrast agent revealed Fallopian tubes of uneven thickness, partially slim. In addition, a semiannular high echoic area was observed around the ovaries, and a small number of microbubbles were dispersed within the pelvic cavity.
3. Tube blocked: the patient was obviously in pain following pressurized infusion. We were unable to see the entire passage of the tube or spillage at the fimbriae end. There was a lack of high echoic areas around the ovaries. There was also a lack of microbubble echo within the pelvic cavity.

### 2.5. Diagnostic Criteria for B Mode-HyCoSy [9, 18]

1. Tube patent: B mode revealed a bright band within the tube. High echoic microbubbles spread rapidly and evenly and repeatedly flowed from the uterus horn to the fimbrial end of the tube. We referred to this sign as “turbulent flow.” The wall of the tube was displayed clearly and was smooth. High echoic microbubbles dispersed in the end of the fimbria.
2. Oviduct passable but not smooth: B mode revealed small amounts of high echoic microbubbles flowing slowly. The full or sectional oviduct presented with a defective and nonhomogenous filling. In addition, a small number of high echoic microbubbles were dispersed in the end of the tube.
3. Oviduct occlusion: failure to display a full or partial bright band and no microbubble echo dispersed at the fimbriae end of the tube.

### 2.6. Diagnostic Criteria for Laparoscopy and Dye Test [17]

1. Tube patent: no resistance during injection and overflow of methylene blue from the Fallopian fimbria.
2. Tube obstruction: apparent resistance during injection, obvious reflux, and the absence of methylene blue overflowing from the Fallopian fimbria.

### 2.7. Evaluation of Discomfort or Pain [19]

Discomfort/pain scoring was as follows: 0 (no reaction or discomfort); 1 (slight pain, less than menstrual pain); 2 (moderate pain, exceeding menstrual cramps but no vagal effects); 3 (vagal effects or pain requiring observation in a hospital); and 4 (vagal effects or pain requiring resuscitation).

### 2.8. Statistical Analysis

Data were analyzed using SPSS, version 19 (SPSS, Chicago, IL, USA). The sensitivity, specificity, and positive (PPV) and negative (NPV) predictive values of the 4D technique were calculated with respect to data arising from laparotomy and dye. Agreement between the two methods was compared using the kappa index value, with kappa >0.75 indicating high consistency.

## 3. Results

In total, 739 female patients were recruited. The age range was 21–44 years and the duration of infertility was 3.2 ± 2.1 years. The examination was conducted during days 5–12 of the menstrual cycle. The patients recruited did not have any history of serious diseases and contraindications. All tests were performed with SonoVue successfully [739 of 739 (100%)]. Six of the 739 women had only one Fallopian tube; 145 had pathological findings in their Fallopian tubes during HyCoSy. Overall, 34 of these 145 cases (62 tubes) were verified using the gold standard laparoscopy and dye test. We defined the segment which was more than 3 cm distal from the uterus as the distal part of the Fallopian tube [20].

**Figure 1:**
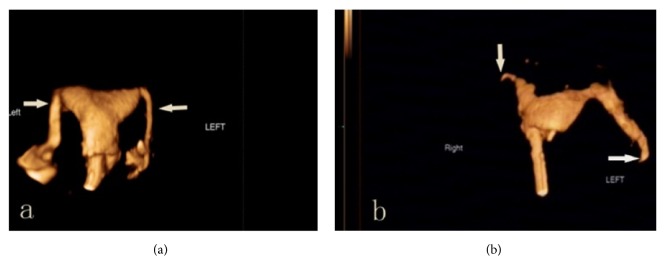
Tubal patency diagnosed by 4D-HyCoSy: (a) bilateral patent oviducts; (b) bilateral obstructed oviduct.

Compared with the laparoscopy and dye test, tubal occlusion diagnostic accordance rates for 4D-HyCoSy were 88.7%(23+32)/62, with a kappa coefficient of 0.769 and a 76.9% agreement rate (Table 1). Distal occlusion diagnostic accordance rates for 4D-HyCoSy were 100% (8/8), with a k coefficient of 1.000 and a 100% agreement rate (Table 2). The sensitivity, specificity, PPV (Positive Predictive Value), and NPV (Negative Predictive Value) of 4D-HyCoSy compared to laparoscopy were 88.4%, 88.8% 85.1%, and 91.4%, respectively (Figure 1).

**Figure 2:**
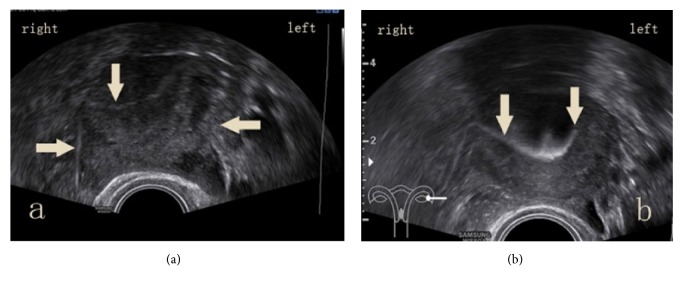
Tubal patency diagnosed by B mode-HyCoSy: (a) right patent oviduct; left patent but not smooth; (b) right patent oviduct; left obstructed oviduct.

Twenty tubes were diagnosed as “patent” by 4D-HyCoSy although the B mode-HyCoSy procedure showed these tubes as passable but not smooth (Figure 2). Four tubes were misdiagnosed as proximal partial obstruction by 4D-HyCoSy, while subsequent B mode-HyCoSy indicated that these tubes were “patent”.

The mean total examination time was 26.2 ± 10 min (range, 9–47 min), and the time taken for the 4D procedure (examination time after intubation) was 5.6 ± 3.5 min (range, 2–18 min), with 42 ± 26 s (range, 12–51 s) for the 4D-HyCoSy examination. Examination time was 11.8 ± 3.5 min (range 3.5–28.5 min) for B mode. The most important factors affecting the length of the examination was likely to be the proficiency of the sonographer and the time taken to insert the catheter.

During HyCoSy, the pain score was 0 in 270 women (36.50%), 1 in 387 women (52.30%), 2 in 50 women (0.06%), 3 in 30 women (0.04%), and 4 in 2 women (0.002%). Thirty patients presented with a severe vasovagal reaction that required observation in hospital but was relieved after 2 h without any medication. Two patients required hospital admission. No infections were observed after the examination (Table 3).

## 4. Discussion

Obstruction of the Fallopian tube may prevent fertilization and thus result in infertility. However, identifying obstructions in the Fallopian tube is a key problem when investigating female infertility in the clinic. HyCoSy has therefore been suggested as a screening method for tubal occlusion to be included in standard infertility tests [18]. With regard to tubal patency, we observed good consistency between the HyCoSy findings and data arising from laparoscopy with dye. A recent study showed that the concordance rate was 90% when HyCoSy was compared to laparoscopy [13]. In this study, we demonstrated that 4D-HyCoSy successfully identified 23 obstructed Fallopian tubes, which were also confirmed by the laparoscopy and dye test, with a sensitivity of 88.4% and a specificity of 88.8%.

**Figure 3:**
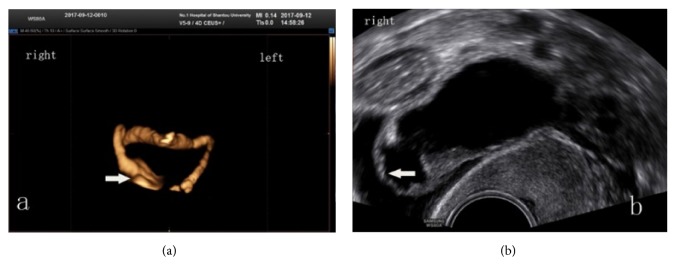
The whole right Fallopian tube appeared to be patent when viewed by 4D-HyCoSy; however, the right distal end of the tube was distended with hydrosalpinx (a). A subsequent examination with B mode-HyCoSy confirmed the existence of an obstruction in the Fallopian tube fimbria (b).

The application of 4D-HyCoSy using SonoVue contrast overcomes some of the limitations and difficulties associated with evaluating tubal patency. The 4D-HyCoSy is a dynamic feature of 2D ultrasound and presents data in 3D which can be done completely in any scanning plane. This procedure can reduce the requirement for a skilled sonographer. In traditional methods, the sonographer has to manipulate the TVS probe quickly in order to detect contrast agent echoes in different sections of the tubes. Moreover, using a high bubble concentration of contrast agent allowed us to distinguish the tubes from surrounding bowel. The 4D-HyCoSy using SonoVue provided an abundance of information with which to evaluate and easily identified the morphology of the Fallopian tubes, as well as the movement of contrast media flowing in the Fallopian tubes and pelvic cavity. Furthermore, the acquired data could be saved for offline analysis and can be reconstructed in any scanning plane [21]. Although 4D-HyCoSy has many advantages, misdiagnosis can still occur due to venous reflux, spasms in the oviducts, dispersion in the pelvis, or inappropriate treatment of the images (Figure 3). Real-time three-dimensional imaging uses a coronal section, and the structure of the same coronal position can overlap each other. The consequence of this is that difficulty in differentiating veins and tubes may occur [22]. We also misdiagnosed the tubes as “patent” when we detected a small amount of microbubbles overflowed from the Fallopian fimbriae after pressurized injection. Finally, we confirmed that the contrast medium near the Fallopian fimbriae was dispersed from the contralateral patent tube. We designed the procedure for B mode-HyCoSy in our study to overcome the limitations of 4D-HyCoSy. B mode ultrasound is able to display the structure of the uterine cavity and trace the strong echo of SonoVue microbubbles passed into the Fallopian tube, which originate from the uterine horn and flowed to the distal end; in contrast, signals from blood vessels and the bowel did not follow this pattern. In addition, signals are weaker due to reverberations which originate from blood vessels and bowel contents.

**Figure 4:**
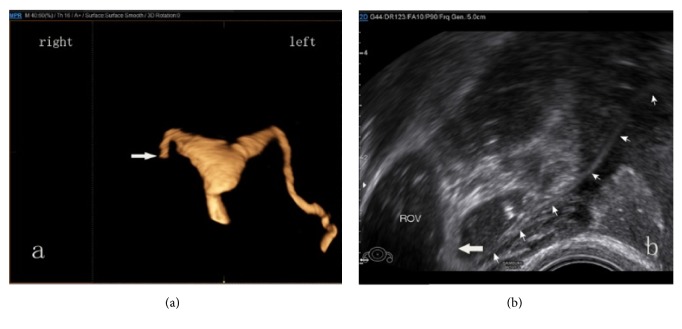
Right oviduct misdiagnosed as proximal partial obstruction in 4D-HyCoSy (a). A subsequent examination with B mode- HyCoSy confirmed the misdiagnosis (b). The whole right Fallopian tube (small arrow); the contrast medium spayed from the Fallopian tube fimbria (large arrow).

Some previous studies have reported that if the obstruction occurs in the middle or distal segment of the Fallopian tubes, then a pathological condition always develops. The detection of contrast flow in the middle and distal sections of the tubes boosted the confidence of sonographers in terms of deeming the tube as being patent. However, the pathological conditions of the proximal part of the Fallopian tube need to be carefully distinguished from transient spasms, thick endometrium, or plugs of mucous [8, 23]. In our study, the diagnosis of distal obstruction in eight Fallopian tubes was consistent with laparoscopic dye. A recent study showed that repeating HyCoSy within a few minutes may rule out false occlusion [24]. Four tubes were misdiagnosed as proximal partial obstruction in 4D-HyCoSy. However, after 2 min, a subsequent examination with B mode- HyCoSy confirmed the misdiagnosis (Figure 4). We believe that the reason for this discrepancy was transient spasms.

Although 4D-HyCoSy can visualize the entire Fallopian tube, it is not capable of accessing the endosalpinx intuitively, while B mode-HyCoSy can. When salpingitis occurs, the endosalpinx becomes gradually irregular and distorted. Furthermore, the papillary excrescences, and the organized granulation tissue protrudes into the surface of the endosalpinx [25]. In our study, 10 cases (20 tubes) were diagnosed as “patent” by 4D-HyCoSy while the B mode-HyCoSy procedure showed a defective and nonhomogenous filling and diagnosed as “patent but not smooth”. Three of these 10 cases suffered Fallopian pregnancy several months after the procedure. We suggest that inflammation destroyed the Fallopian tube wall, resulting in weakening of peristalsis, integrity of ciliate epithelium, and ciliate activity.

In a previous study, Chiara et al. reported that even the “tubal patency” does not mean that the oviducts have normal functionality. The clinical meaning of “tubal factor” includes more. Tubal peristalsis and normal ciliate epithelium are an absolute necessity to permit fertilization and implantation [26]. In B mode-HyCoSy procedure, we observed an interesting sign, which we referred to as “turbulent flow” [20] in which high echoic microbubble masses moved from place to place in the intramural part of the tube in an even and repetitive manner. We speculate that “turbulent flow” is an indirect assessment of tubal peristalsis and that this sign might imply reliability of tubal patency testing and the normal function of the tube.

## 5. Conclusions

We suggest that B mode-HyCoSy is an additional technique for 4D-HyCoSy and the combination of both methods may improve diagnostic accuracy. These procedures should be understood as being complementary rather than competitive. The drawback of our study was that we did not perform laparoscopic dye testing in normal Fallopian tubes, so we could not presume that HyCoSy was an accurate test for evaluating the overall health of the Fallopian tube. All patients had very low pain scores during the test, and only 2 cases (2/739, 0.002%) developed complications while undergoing HyCoSy and required resuscitation.

## Figures and Tables

**Table 1:** Agreement between hysterosalpingo-contrast sonography (HyCoSy) and the laparoscopic dye (LD) test.

		LD	
HyCoSy	patent	occlusion	total
patent	32	3	35
occlusion	4	23	27
total	36	26	62
	Kappa:0.769		

**Table 2:** Agreement of distal occlusion between hysterosalpingo-contrast sonography (HyCoSy) and the laparoscopic dye (LD) test.

		LD	
HyCoSy	patent	distal occlusion	total
patent	8	0	8
distal occlusion	0	8	8
total	8	8	16
	Kappa:1.000		

**Table 3:** Adverse reactions of the patients during HyCoSy and the distribution of ranks.

Classifications (ranks)	Clinical feature	number	percentage
0	no reaction or discomfort	270	36.5%
1	slight pain, less than menstrual pain	387	52.3%
2	moderate pain, exceeding menstrual cramps but no vagal effects	50	0.067%
3	vagal effects or pain requiring observation in a hospital	30	0.04%
4	vagal effects or pain requiring resuscitation	2	0.002%

## Data Availability

The data used to support the findings of this study are available from the corresponding author upon request.
